# Factors associated with antepartum pilgrimage at a reference maternity hospital in Ceará

**DOI:** 10.1590/1980-220X-REEUSP-2023-0012en

**Published:** 2024-04-12

**Authors:** Annita de Lima Mesquita, Hillary Bastos Vasconcelos Rodrigues, Uly Reis Ferreira, Maria Aparecida Ferreira Domingos, Bruno Luciano Carneiro Alves de Oliveira, Alexandrina Maria Ramos Cardoso, Camila Biazus-Dalcin, Priscila de Souza Aquino

**Affiliations:** 1 Universidade Federal do Ceará, Departamento de Enfermagem, Fortaleza, CE, Brazil.; 2 Universidade Federal do Maranhão, Departamento de Medicina I, São Luís, MA, Brazil.; 3 Universidade do Porto, Escola de Enfermagem do Porto, Porto, Portugal.; 4 University of Dundee, School of Health Sciences, Dundee, Scotland.

**Keywords:** Health Services Accessibility, Birthing Centers, Parturition, Barriers to Access of Health Services, Women’s Health, Accesibilidad a los Servicios de Salud, Centros de Asistencia al Embarazo y al Parto, Parto, Barreras de Acceso a los Servicios de Salud, Salud de la Mujer, Acessibilidade aos Serviços de Saúde, Centros de Assistência à Gravidez e ao Parto, Parto, Barreiras ao Acesso aos Cuidados de Saúde, Saúde da Mulher

## Abstract

**Objective:**

To identify factors associated with antepartum pilgrimage in pregnant women in Fortaleza, Ceará, Brazil.

**Method:**

A cross-sectional study with 300 postpartum women from a state reference maternity hospital, carried out from March 2020 to January 2021. The frequency of pilgrimage was estimated according to socioeconomic characteristics and prenatal care. Analysis with Pearson’s chi-square test selected variables for adjusted Poisson regression.

**Results:**

The frequency of antepartum pilgrimage to more than one health service was 34.3%. Not knowing the reference maternity hospital (1.16; 95%CI: 1.04–1.30) and not living close to the reference maternity hospital (1.16; 95%CI: 1.03–1.31) were associated with the occurrence of pilgrimage among women. Personal characteristics and prenatal care were not associated.

**Conclusion:**

There was an association between antepartum pilgrimage and lack of knowledge of the reference maternity hospital and residence far from that maternity hospital, which requires better team communication and the guarantee of easier access to obstetric care services, through effective implementation of regionalization of maternal care.

## RESUMO

**Objetivo:**

Identificar fatores associados à peregrinação anteparto em gestantes de Fortaleza, Ceará, Brasil.

**Método:**

Estudo transversal com 300 puérperas de uma maternidade de referência estadual, realizado de março de 2020 a janeiro de 2021. Foi estimada a frequência da peregrinação segundo características socioeconômicas e de assistência pré-natal. Análises com o Teste Qui-Quadrado de Pearson selecionaram variáveis para a regressão de Poisson ajustada.

**Resultados:**

A frequência de peregrinação anteparto por mais de um serviço de saúde foi de 34,3%. Não conhecer a maternidade de referência (1,16; IC95% 1,04–1,30) e não residir próximo à maternidade de referência (1,16; IC95%: 1,03–1,31) estiveram associados à ocorrência de peregrinação entre as mulheres. Características pessoais e de assistência pré-natal não se mostraram associadas.

**Conclusão:**

Verificou-se associação da peregrinação anteparto com o desconhecimento da maternidade de referência e com a residência distante dessa maternidade, o que requer melhor comunicação da equipe e a garantia de acesso facilitado aos serviços de assistência obstétrica por meio da implementação eficaz da regionalização dos cuidados maternos.

## RESUMEN

**Objetivo:**

Identificar factores asociados a la peregrinación anteparto en mujeres embarazadas en Fortaleza, Ceará, Brasil.

**Método:**

Estudio transversal con 300 puérperas de una maternidad de referencia estatal, realizado de marzo de 2020 a enero de 2021. Se estimó la frecuencia de peregrinación según características socioeconómicas y de atención prenatal. Análisis con Prueba de Chi-Cuadrado de Pearson variables seleccionadas para regresión de Poisson ajustada.

**Resultados:**

La frecuencia de peregrinación anteparto a más de un servicio de salud fue del 34,3%. Desconocer la maternidad de referencia (1,16; IC95%: 1,04–1,30) y no vivir cerca de la maternidad de referencia (1,16; IC95%: 1,03–1,31) se asociaron con la ocurrencia de peregrinación entre las mujeres. Las características personales y la atención prenatal no se asociaron.

**Conclusión:**

Hubo asociación entre la peregrinación anteparto y el desconocimiento de la maternidad de referencia y vivir lejos de esa maternidad, lo que requiere una mejor comunicación del equipo y garantizar un acceso más fácil a los servicios de atención obstétrica a través de la implementación efectiva de la regionalización de la atención materna.

## INTRODUCTION

Assistance to pregnant women is the main modulator of outcomes related to maternal and child health. The longitudinality of care is guaranteed through prenatal appointments that provide bonding, health promotion, disease prevention and user satisfaction^(1)^. However, prenatal care is provided in Primary Health Care Units and outpatient services that are not always adequately linked to reference maternity hospitals for obstetric emergencies and childbirth^(2)^.

Therefore, antepartum pilgrimage, which consists of the search undertaken by pregnant women for multiple health establishments in search of assistance during childbirth, when these places do not have places for their hospitalization, may result from the absence of a prior link with the reference maternity hospital^(3,4)^. This reflects potential risks to the mother/baby dyad’s health, highlighting maternal and neonatal mortality, which reduction is one of the Sustainable Development Goals of the United Nations^(3,5)^.

Obstetric care is permeated by multiple barriers that affect access and use of this care. The wide territorial extension of Brazil favors organizational inequality in health units, resulting in inadequate access, resulting in different geographic scenarios^(6)^. Despite Law 11,634/2007 ensuring women have prior knowledge of the maternity hospital where they will give birth, pilgrimages still occur. A study with 768 women from Sergipe showed that 29.4% had an antepartum pilgrimage^(4)^. Furthermore, the Stork Network Strategy highlights the need to strengthen the Health Care Network guidelines and integrated regional planning, in order to guarantee access to health services^(7)^.

This pilgrimage during labor constitutes one of the most common obstetric violence in our country. According to a study carried out in public maternity hospitals in the Northeast, the majority of women interviewed defined the search for a maternity hospital as a traumatic event. Among the reasons for pilgrimage are, mainly, the limitations of material resources and overcrowding, demonstrating that the bed regulation system is flawed, and sometimes fails to make adequate referrals^(8)^.

Considering the above, it is essential to understand the factors that influence antepartum pilgrimage, in order to identify the vulnerabilities that exist in the female population. Furthermore, these factors are not yet as clear as possible due to few studies addressing the topic. This knowledge can support specific public policies and guide professional practices. In view of this problem, this article aims to identify the factors associated with antepartum pilgrimage in pregnant women in Fortaleza, Ceará, Brazil.

## METHOD

### Study Design

This is an evaluative, sectional study. The writing of the article followed the STrengthening the Reporting of OBservational studies in Epidemiology (STROBE) protocol instructions^(9)^.

### Place

This study was carried out in the rooming-in of a reference maternity hospital in the state of Ceará, located in Fortaleza, which receives patients from all over the state, performing habitual and high-risk births.

### Population and Selection Criteria

The study population was postpartum women hospitalized in rooming-in. As inclusion criteria, it was adopted to be in the immediate postpartum period with a live newborn, to have had a high-risk pregnancy and to have undergone follow-up in primary care. The exclusion criteria were having undergone a period of hospitalization during pregnancy, compromising access to usual prenatal care.

### Sample Definition

To calculate the finite sample, a confidence level of 95%, a number of annual births of 4,000 and a sampling error of 5% were considered. The sample consisted of 300 women, selected according to entry into the unit.

### Data Collection

Data collection began in March 2020, ending in January 2021. A structured form was used with sociodemographic, obstetric and access to health services data, created by the study authors. The outcome was pilgrimage, defined as the search for more than one health service to assist during labor. The predictor variables were age, origin, race, living with a partner, education, income (in minimum wages), gestational age at the start of prenatal care (weeks), number of prenatal appointments, companion at appointments, delay in appointment scheduling, prenatal care provided by nurses, knowledge of reference maternity hospital, residence close to reference maternity hospital, travel time to reference maternity hospital and difficulty in traveling to reference maternity hospital.

The interviews were carried out directly with the women, in the morning or afternoon, in their own bed, recorded on a paper form, with an average duration of 15 minutes. Data from pregnant women’s record were collected for prenatal access information.

### Data Analysis and Processing

The data from the present study were tabulated using the Statistical Package for the Social Sciences (SPSS) version 22.0. The frequency of pilgrimage was estimated according to sociodemographic characteristics and prenatal care. Statistical differences were identified by Pearson’s chi-square test, with a significance level of 5% (type I error). Then, variables that showed an association were included in the unadjusted and adjusted Poisson regression model (one variable for the other) to estimate Prevalence Ratio (PR) and 95% Confidence Interval (95%CI).

### Ethical Aspects

The study was approved by the *Maternidade Escola Assis Chateaubriand* (MEAC) Research Ethics Committee, under Protocol 3.673.810.

To apply the chosen instrument, postpartum women were informed about the research, and those who agreed to participate signed the Informed Consent Form (ICF). Underage pregnant women who agreed to participate in the study signed the Assent Form, and their guardians signed the ICF.

## RESULTS

A total of 300 postpartum women participated in the study. Of these, 34.3% (n = 103) sought more than one health service to deliver the baby. In Table 1, data relating to the frequency of antepartum pilgrimage of postpartum women were displayed according to sociodemographic variables.

**Table 1:** Association of sociodemographic variables with antepartum pilgrimage of postpartum women – Fortaleza, CE, Brazil, 2021.

Variables	Antepartum pilgrimage				p-value*
	Yes		No		
	n	(%)	n	(%)	
**Age (years)**					0.101
Up to 19	29	(42.6)	39	(57.4)	
≥20	74	(31.9)	158	(68.1)	
**Origin**					**0.18**
Capital	70	(30.7)	158	(69.3)	
Others	33	(45.7)	39	(54.3)	
**Black race**					0.363
Yes	5	(25.0)	15	(75.0)	
No	98	(35.0)	182	(65.0)	
**Living with partner**					0.560
Yes	78	(33.5)	155	(66.5)	
No	25	(37.3)	42	(62.7)	
**Education (years)**					0.259
Up to 9 years	44	(38.3)	71	(61.7)	
Over 9 years	59	(31.9)	126	(68.1)	
**Income (wage)**					0.274
Up to 1 wage	56	(37.3)	94	(62.7)	
Above 1 wage	47	(31.3)	103	(68.7)	

Note: *Pearson’s chi-square test.

The origin variable showed a significant p-value (p = 0.018) with the investigated outcome. The variables age, black race, education, living with a partner, education and income were not statistically significant.

Table 2 presents the frequency of antepartum pilgrimage of postpartum women according to prenatal care variables.

**Table 2:** Association of characteristics of prenatal care and access with antepartum pilgrimage of postpartum women – Fortaleza, CE, Brazil, 2021.

Variable	Pilgrimage		p-value*
	Yes	No	
	n (%)	n (%)	
**Start of prenatal care (weeks)**			
Under 12	57 (19.0)	89 (29.7)	0.095
Over 12	46 (15.3)	108 (36.0)	
**Number of prenatal care appointments**			
Less than 7	35 (33.3)	70 (66.7)	0.789
7 or more	68 (34.9)	127 (65.1)	
**Companion to prenatal care appointments**			
Yes	61 (33.7)	120 (66.3)	0.776
No	42 (35.3)	77 (64.7)	
**Delay in scheduling appointments**			
Yes	22 (37.9)	36 (62.1)	0.521
No	81 (33.5)	161 (66.5)	
**Prenatal care carried out by a nurse**			
Yes	101 (34.2)	194 (65.8)	0.788
No	2 (40.0)	3 (60.0)	
**Knowledge of RMH** ^1^			
Yes	52 (27.7)	136 (72.3)	**0.001**
No	50 (46.7)	57 (53.3)	
**Residence close to RMH** ^1^			
Yes	22 (19.5)	91 (80.5)	**<0.001**
No	81 (43.3)	106 (56.7)	
**Travel time to RMH** ^1^			
Up to 30 minutes	40 (24)	127 (76.4)	**<0.001**
Over 30 minutes	39 (41.1)	56 (58.9)	
**Difficulty moving to RMH** ^1^			
Yes	28 (80.0)	7 (20.0)	**<0.001**
No	75 (28.4)	189 (71.6)	

Note: *Pearson’s chi-square test;

^1^Reference maternity hospital.

Regarding prenatal care and access characteristics, an association of the following variables was evident: knowledge of reference maternity hospital (p = 0.001); residence close to reference maternity hospital (p < 0.001); and travel time of up to 30 minutes.

Table 3 presents the Poisson regression model with robust variance.

**Table 3:** Poisson regression with robust unadjusted and adjusted variance – Fortaleza, CE, Brazil, 2021.

Variables	Raw analysis		Adjusted analysis	
	PR	95%CI	PR	95%CI
**Origin**				
Outside the capital	1.49	1.08–2.05	1.06	0.92–1.21
Capital	1	–	1	–
**Knowledge of reference maternity hospital**				
No	1.68	1.24–2.29	1.16	1.04–1.30
Yes	1	–	1	–
**Residence close to reference maternity hospital**				
No	2.22	1.47–3.35	1.16	1.03–1.31
Yes	1	–	1	–
**Travel time over 30 minutes**				
Yes	1.97	1.43–2.73	1.10	0.97–1.26
No	1	–	1	–
**Difficulty traveling to maternity hospital**				
Yes	2.81	2.18–3.62	1.09	0.94–1.26
No	1	–	1	–

Note: RP – Prevalence Ratio; CI – Confidence Interval.

As seen in Table 3, the association between not knowing the reference maternity hospital and not living close to it remained, with a 16% greater chance for these predictors.

## DISCUSSION

According to the results presented, a percentage of 34.3% of antepartum pilgrimage was observed in the studied population, supporting the recent national survey that showed the Northeast region with a 33.1% rate of pilgrimage during childbirth, the highest rate as well as largest region with pilgrimage in two hospitals (5.1%)^(10)^. Similarly, another study, carried out in two Brazilian cities, São Luís (Northeast) and Ribeirão Preto (Southeast), showed discrepant antepartum pilgrimage rates in the regions: 35.8% and 5.8%, respectively^(11)^. Therefore, attention should be paid to the significant differences in percentages of antepartum pilgrimage between Brazilian regions.

The obstetric care network structuring is an essential condition for adequate assistance and part of the principles of regionalization and decentralization of the Brazilian Health System. Municipalities in the countryside need to have the capacity to resolve habitual risk births, in order to not overload the network of reference maternity hospitals^(12)^. In the USA, high-level hospitals are located in more densely populated areas, and it is important to note that this type of service needs to have care compatible with the level of complexity. To advance perinatal regionalization research, knowledge of associated patient outcomes, and optimize provision of risk-appropriate care, alternative approaches to determining the level of maternal care provided in hospitals are needed^(13)^.

In the present study, not knowing the reference maternity hospital increased the occurrence of antepartum pilgrimage by 16%. In Brazil, since 2007, Law 11,634 guarantees pregnant women the knowledge and connection to the reference maternity hospital for emergency care and childbirth care^(14)^, and the current maternal and child health policy also highlights the importance of this connection^(7)^. A study carried out with 319 postpartum women showed that lack of guidance on finding a reference hospital resulted in 25% inefficiency in prenatal care quality, highlighting failure to comply with current legislation^(15)^.

This problem is recurrent and, regarding the provision of guidance to pregnant women during prenatal care about bonding maternity and the possibility of a prior visit, a study carried out in southern Brazil observed that 44.9% of those seen by a doctor and nurse together received this guidance. Meanwhile, in those attended only by a doctor, the frequency was only 35.6%, which highlights the importance of nursing in providing this information, although in the present study this service was not associated with the occurrence of pilgrimage^(16)^.

In a study carried out with 12 pregnant women in southern Brazil, it was identified that none of them were informed about motherhood or referred; this fact can favor pilgrimage for services as well as contribute to unfavorable outcomes for the mother-fetus dyad^(17)^. Research carried out in Sergipe, a state in northeastern Brazil, with 768 postpartum women, found that prenatal care was a protective factor for pilgrimage, as women who were advised during prenatal care about their reference maternity hospital made fewer pilgrimages^(2)^.

Therefore, the importance of this guidance in prenatal appointments is highlighted to promote prior knowledge of reference maternity hospital. Furthermore, the relevance of maintaining an organized and integrated care network is reinforced, with the aim of reducing pilgrimage of pregnant women to their respective reference maternity hospitals.

These actions can favor the experience of a positive pregnancy and childbirth experience, defined as physical and sociocultural normality, an effective transition to labor and childbirth and positive motherhood, which has been listed as a priority, included in global obstetric health care agendas, with high evidence of recommendation^(18)^.

A study carried out using birth data from Recife, Brazil, showed that, in the state capital, there were 1.5 times the number of births expected for the year, 56% of which were non-resident mothers. On the other hand, no maternity hospital in the countryside responded to the expected volume of usual risk births, causing overcrowding in maternity hospitals in Recife^(12)^. This condition can generate a pilgrimage of women referred to this health unit, highlighting gaps in countryside women’s knowledge about the reference maternity hospital or even flaws in agreements within the care network.

International studies have highlighted the association between physical barriers to access and use of obstetric health care equipment^(19,20)^. A study with 6,655 African women (Burkina Faso) showed that those who lived further away were significantly less likely to give birth in a health unit^(19)^. These data are consistent with the findings of this study, since not living close to the reference maternity hospital increased the chance of antepartum pilgrimage by 16%.

A systematic review carried out with 31 studies showed that having access to obstetric care facilities within a radius of five kilometers was significantly associated with institutional births (pooled OR = 2.27; 95% CI = 1.82, 2.82). Similarly, a travel time of 60 minutes or less was significantly associated with greater odds of delivery in a health care facility (pooled OR = 3.30; 95% CI = 1.97, 5.53). On the other hand, an increase of one hour in travel time and one kilometer in distance was negatively associated with the use of institutional birth care^(21)^.

Thus, distance from services and travel time are important factors associated with adequate obstetric care and may be related. Distance may result in longer travel time. However, geographic barriers, such as delta areas, can also interfere. Travel time to the maternity hospital is a factor cited in the literature as important for carrying out adequate care management in a timely manner, reducing bad prognoses^(22)^. Greater distances and travel time may be associated with births that occur during the maternity hospital^(23)^ as well as adverse birth outcomes, such as the need for admission to Intensive Care Units, postpartum transfusion, post-cesarean hysterectomy, among others^(24)^. In the USA, a cohort study carried out with a total of 662,245 birth records showed that greater distances to the birth hospital were associated with a greater risk of adverse maternal outcomes and admission to the Neonatal Intensive Care Unit^(25)^.

Other studies have linked travel time and distance to the maternity hospital with perinatal outcomes^(26,27)^. Although no significant association with distance was found, the odds of stillbirth were significantly higher for trips of 10 to 29 minutes (OR 2.25, 95% CI 1.40 to 3.63), 30 to 59 minutes (OR 2 .30, 95% CI 1.22 to 4.34) and 60 to 119 minutes (OR 2.35, 95% CI 1.05 to 5.25)^(28)^. Meta-analysis on the relationship between distance and use of health services in Sub-Saharan Africa identified that increasing distance to the maternity hospital had an inverse association with use. The distance from a hospital for rural women showed an even more pronounced effect on use, showing that the difficulty of geographic access prevents use^(29)^.

In a study carried out on the border between Thailand and Myanmar, the distance traveled to the clinic strongly predicted loss of follow-up (these traveled 50% longer), miscarriage (these traveled 20% longer), malaria infections during pregnancy (covered a 60% longer distance) and presentation for prenatal care after the first trimester (these traveled a 50% longer distance). This analysis provided the first evidence of the complex impact of geography on access to prenatal services and pregnancy outcomes in the rural, remote and politically complex Thai-Myanmar border region^(30)^.

Therefore, living close to the maternity hospital is actually beneficial for maternal and child care. It is clear that accessibility to obstetric care services involves proximity to the reference maternity hospital as well as knowledge of it, which requires implementing health policies that already consider these aspects as guidelines.

As limitations of this study, there is memory bias due to participants’ self-declaration and collection taking place at the time of admission to the maternity hospital, with postpartum women constantly paying attention to newborns, which resulted in some interruptions in the interview.

## CONCLUSION

The present study found a significant percentage of pilgrimage in the studied population, with associated factors being lack of knowledge of reference maternity hospital and not living close to it.

Proper guidance to pregnant women about the obstetric care network still represents a challenge in ensuring quick access to this service. Thus, the study reinforces the importance of implementing current health policies in a context in which organization of services still presents weaknesses. The greater commitment of health professionals in guiding pregnant women about their reference maternity hospital is important in order to prevent pilgrimage and its consequences to the dyad.

Therefore, to minimize pilgrimage, health policies need to effectively guarantee access to obstetric care services, with timely delivery, through the effective implementation of maternal care regionalization, with the care network organization coordination.
